# Supplementary material for: Heat adaptation of phage T7 under an extended genetic code
Source: Virus Evol. 2021 Dec 1;7(2):veab100. doi: 10.1093/ve/veab100 (PMC8923235; doi:10.1093/ve/veab100)
Supplement: veab100_Supp [file veab100_supp.zip › Sequencing_Analysis.zip!/Sequencing_Analysis/S2/evidence/JC_55.html]

BRESEQ :: Evidence


| New junction evidence | | | | | | | | | | | |
| --- | --- | --- | --- | --- | --- | --- | --- | --- | --- | --- | --- |
|  | | seq id | position | reads (cov) | reads (cov) | score | skew | freq | annotation | gene | product |
| \* | ? | NC\_001604.1 | = 8335 | 5558 (1.050) | 25 (0.000) | 22/584 | 44.4 | 0.45% | intergenic (–/–) | *–/–* | –/– |
|  | ? | NC\_001604.1 | 8939 = | 5643 (1.100) |  |  |  |  | intergenic (–/–) | *–/–* | –/– |
| Rejected: Coverage evenness skew score above cutoff. | | | | | | | | | | | |
| Rejected: Frequency below/above cutoff threshold. | | | | | | | | | | | |

Only 100 of 257 total aligned reads displayed.  

`GGCACCGAGCGTCCACATCATTATCACCCGTGGTGACTTTGAGAAAGCAATAGACAAGAAACGTCCGGTTCTTAGTGTGGCAGTGACCCGCTTCCCGTTCGTCCGTCTGTTACTCAAACGAATCAAGGAGGTGTTCTGATGGGACTGTTAGATGGTGAAGCCTGGGAAAAAGAAAACCCGCCAGTACAAGCAACTGGGTGTATAGCTTGCTTAGAGAAAGATGACCGTTATCCACACACCTGTAACAAAGGAGCTAACGATATGACCGAACGTG‑AACAAGAGATGATCATTAAGTTGATAGACAATAA‑‑‑‑‑‑‑‑‑‑‑‑‑‑‑‑‑‑‑‑‑‑‑‑‑‑‑‑‑‑‑‑‑‑‑‑‑‑‑‑‑‑‑‑‑‑‑‑‑‑‑‑‑‑‑‑‑‑‑‑‑‑‑‑‑‑‑‑‑‑‑‑‑‑‑‑‑‑‑‑‑‑‑‑‑‑‑‑‑‑‑‑‑‑‑‑‑‑‑‑‑‑‑‑‑‑‑‑‑‑‑‑‑‑‑‑‑‑‑‑‑‑‑‑‑‑‑‑‑‑‑‑‑‑‑‑‑‑‑‑‑‑‑‑‑‑‑‑‑‑‑‑‑‑‑‑‑‑‑‑‑‑‑‑‑‑‑‑‑‑‑‑‑‑‑‑‑‑‑‑‑‑‑‑‑‑‑‑‑‑‑‑‑‑‑‑‑‑‑‑‑‑‑‑‑‑‑‑‑‑‑‑‑‑‑‑‑‑‑‑‑‑‑‑‑‑‑‑‑‑‑‑‑‑‑‑‑‑‑‑‑‑‑‑‑‑‑‑‑‑‑‑‑‑‑‑‑‑‑‑‑‑‑‑‑‑‑‑‑‑‑‑‑‑‑‑‑‑‑‑‑‑‑‑‑‑‑‑‑‑‑‑‑‑‑‑‑‑‑  >  NC_001604.1/8028‑8335`  
`‑‑‑‑‑‑‑‑‑‑‑‑‑‑‑‑‑‑‑‑‑‑‑‑‑‑‑‑‑‑‑‑‑‑‑‑‑‑‑‑‑‑‑‑‑‑‑‑‑‑‑‑‑‑‑‑‑‑‑‑‑‑‑‑‑‑‑‑‑‑‑‑‑‑‑‑‑‑‑‑‑‑‑‑‑‑‑‑‑‑‑‑‑‑‑‑‑‑‑‑‑‑‑‑‑‑‑‑‑‑‑‑‑‑‑‑‑‑‑‑‑‑‑‑‑‑‑‑‑‑‑‑‑‑‑‑‑‑‑‑‑‑‑‑‑‑‑‑‑‑‑‑‑‑‑‑‑‑‑‑‑‑‑‑‑‑‑‑‑‑‑‑‑‑‑‑‑‑‑‑‑‑‑‑‑‑‑‑‑‑‑‑‑‑‑‑‑‑‑‑‑‑‑‑‑‑‑‑‑‑‑‑‑‑‑‑‑‑‑‑‑‑‑‑‑‑‑‑‑‑‑‑‑‑‑‑‑‑‑‑‑‑‑‑‑‑‑‑‑‑‑‑‑‑‑‑‑‑‑‑‑‑‑‑‑‑‑‑‑‑‑‑‑‑‑‑‑‑‑‑‑‑‑‑‑‑‑‑‑‑‑‑‑‑‑‑‑‑‑‑‑gacaataaGAAGTTTTGGGCTACCGTAGAGTCCTCGGAGCATTCCTTCGAGGTTCCAATCTACGCTGAGACCCTAGACGAAGCTCTGGAGTTAGCCGAATGGCAATACGTTCCGGCTGGCTTTGAGGTTACTCGTGTGCGTCCTTGTGTAGCACCGAAGTAATACGACTCACTATTAGGGAAGACTCCCTCTGAGAAACCAAACGAAACCTAAAGGAGATTAACATTATGGCTAAGAAGATTTTCACCTCTGCGCTGGGTACCGCTGAACCTTACGCTTACATCGCCAAGCCGGACTACGGCAACGA  >  NC_001604.1/8939‑9237`  

                                                                                                                                                                                                                                                                                                                                                                                                                                                                                                                                                                                                                                  
`gagatgtgtataagagacagTTATCACCCGTGGTGACTTTGAGAAAGCAATAGACAAGAAACGTCCGGTTCTTAGTGTGGCAGTGACCCGCTTCCCGTTCGTCCGTCTGTTACTCAAACGAATCAAGGAGGTGTTCTGATGGGACTGTTAGATGGTGAAGCCTGGGAAAAAGAAAACCCGCCAGTACAAGCAACTGGGTGTATAGCTTGCTTAGAGAAAGATGACCGTTATCCACACACCTGTAACAAAGGAGCTAACGATATGACCGAACGTG‑AACAAGAGATGATCATTAAGTTGATAG                                                                                                                                                                                                                                                                                                                    <  1:13951/281‑1`  
`gagacaGAGAGTCCACATCTTTATCACCCGTTGTGACATATAGAAAGCAATAGACAAGAAACGTCCGGTTCTTAGTGTGGCAGTGACCCGCTTCCCGTTCGTCCGTCTGTTACTCAAACAAATCAAGGAGGTGTTCTGATGGGACTGTTAGATGGTGAGGCCTGGGAAAAAGAAAACCCGCCAGTACAAGCAACTGGGTGTATAGCTTGCTTAGAGAAAGATGCCCGATATCCACACACCTGTAACAAAGGAGCTAACTATATGACCGAACGTG‑AACAAGAGATGATCATTAAGTTGATAG                                                                                                                                                                                                                                                                                                                    <  1:821600/295‑1`  
`GGCACCGAGCGTCCACATCATTATCACCCGTGGTGACTTTGAGAAAGCAATAGACAAGAAACGTCCGGTTCTTAGTGTGGCAGTGACCCGCTTCCCGTTCGTCCGTCTGTTACTCAAACGAATCAAGGAGGTGTTCTGATGGGACTGTTAGATGGTGAAGCCTGGGAAAAAGAAAACCCGCTAGTACAAGCAACTGGGTGTATAGCTTGCTTAGAGAAAGATGACCGTTATCAACACACCTGTAACAAAGGAGATAACGATATGACCGAAGGTG‑AACAAGAGATGATCATTAAGTTGATAG                                                                                                                                                                                                                                                                                                                    >  1:130574/1‑301`  
`GGCACCGAGCGTCCACATCATTATCACCCGTGGTGACTTTGAGAAAGCAATAGACAAGAAACGTCCGGTTCTTAGTGTGGCAGTGACCCGCTTCCCGTTCGTCCGTCTGTTACTCAAACGAATCAAGGAGGTGTTCTGATGGGACTGTTAGATGGTGAAGCCTGGGAAAAAGAAAACCCGCCAGTACAAGCAACTGGGTGTATAGCTTGCTTAGAGAAAGATGACCGTTATCCACACACCTGTAACAAAGGAGCTAACGATATGACCGAACGTG‑AACAAGAGATGATCATTAAGTTGATAG                                                                                                                                                                                                                                                                                                                    >  1:736263/1‑301`  
`GGCACCGAGCGTCCACATCATTATCACCCGTGGTGACTTTGAGAAAGCAATAGACAAGAAACGTCCGGTTCTTAGTGTGGCAGTGACCCGCTTCCCGTTCGTCCGTCTGTTACTCAAACGAATCAAGGAGGTGTTCTGATGGGACTGTTAGATGGTGAAGCCTGGGAAAAAGAAAACCCGCCAGTACAAGCAACTGGGTGTATAGCTTGCTTAGAGAAAGATGACCGTTATCCACACACCTGTAACAAAGGAGCTAACGATATGACCGAACGTG‑AACAAGAGATGATCATTAAGTTGATAG                                                                                                                                                                                                                                                                                                                    >  1:213373/1‑301`  
`GGCACCGAGCGTCCACATCATTATCACCCGTGGTGACTTTGAGAAAGCAATAGACAAGAAACGTCCGGTTCTTAGTGTGGCAGTGACCCGCTTCCCGTTCGTCCGTCTGTTACTCAAACGAATCAAGGAGGTGTTCTGATGGGACTGTTAGATGGTGAAGCCTGGGAAAAAGAAAACCCGCCAGTACAAGCAACTGGGTGTATAGCTTGCTTAGAGAAAGATGACCGTTATCCACACACCTGTAACAAAGGAGCTAACGATATGACCGAACGTG‑AACAAGAGATGATCATTAAGTTGATAG                                                                                                                                                                                                                                                                                                                    >  1:447465/1‑301`  
`GGCACCGAGCGTCCACATCATTATCACCCGTGGTGACTTTGAGAAAGCAATAGACAAGAAACGTCCGGTTCTTAGTGTGGCAGTGACCCGCTTCCCGTTCGTCCGTCTGTTACTCAAACGAATCAAGGAGGTGTTCTGATGGGACTGTTAGATGGTGAAGCCTGGGAAAAAGAAAACCCGCCAGTACAAGCAACTGGGTGTATAGCTTGCTTAGAGAAAGATGACCGTTATCCACACACCTGTAACAAAGGAGCTAACGATATGACCGAACGTG‑AACAAGAGATGATAATTAAGTTGATAG                                                                                                                                                                                                                                                                                                                    >  1:825139/1‑301`  
 `agagacAGCGTCCACATCATTATCCCCCGTGGTGACTTTGAGAAAGCAATAGACAAGAAACGTCCGGTTCTTAGTGTGGCAGTGACCCGCTTCCCGTTCGTCCGTCTGTTACTCAAACGAATCAAGGAGGTGTTCTGATGGGACTGTTAGATGGTGAAGCCTGGGAAAAAGAAAACCCGCCAGTACAAGCAACTGGGTGTATAGCTTGCTTAGAGAAAGATGACCGTTATCCACACACCTGTAACAAAGGAGCTAACGATATGACCGAACGTG‑AACAAGAGATGATCATTAAGTTGATAGA                                                                                                                                                                                                                                                                                                                   <  1:1115/295‑1`  
 `GCATCGAGCGTCCACATCATTATCACCCGTGGTGACTTTGAGAAAGCAATGGACAAGAAACGTCCGGTTCTTAGTGTGGAAGTGACCCGCTTCCCGTTCGTCCGTCTGTTACTCAAACGAATCAAGGAGGTGTTCTGATGGGATTGTTAGATGGTGAAGCCTGGGAAAAAGAAAACCCGCCAGTACAAGCAACTGGGTGTATAGCTTGCTTAGAGAAAGATGACCGTTATCCACACACCTGTAACAAAGGAGCTAACGATATGACCGAACGTG‑AACAAGAGATGATCATTAAGTTGATAGA                                                                                                                                                                                                                                                                                                                   <  1:509645/301‑1`  
 `GCACTGAGCGTCCACATCATTATCACCCGTGGTGACTTTGAGAAAGCAATAGACAAGAAACGTCCGGTTCTTAGTGTGGCAGTGACCCGCTTCCCGTTCGTCCGTCTGTTACTCAAACGAATCAAGGAGGTGTTCTGATGGGACTGTTAGATGGTGAAGCCTGGGAAAAAGAAAACCCGCCAGTACAAGCAACTGGGTGTATAGCTTGCTTAGAGAAAGATGACCGTTATCCACACACCTGTAACAAAGGAGCTAACGATATGACCGAACGTG‑AACAAGAGATGATCATTAAGTTGATAGA                                                                                                                                                                                                                                                                                                                   >  1:648768/1‑301`  
 `GCACTGAGCGTCCACATCATTATCACCCGTGGTGACTTTGAGAAAGCAATAGACAAGAAACGTCCGGTTCTTAGTGTGGCAGTGACCCGCTTCCCGTTCGTCCGTCTGTTACTCAAACGAATCAAGGAGGTGTTCTGATGGGACTGTTAGATGGTGAAGCCTGGGAAAAAGAAAACCCGCCAGTACAAGCAACTGGGTGTATAGCTTGCTTAGAGAAAGATGACCGTTATCCACACACCTGTAACAAAGGAGCTAACGATATGACCGAACGTG‑AACAAGAGATGATAATTAAGTTGATAGc                                                                                                                                                                                                                                                                                                                   >  1:317413/1‑300`  
 `GCACCGAGCGTCCACATCATTATCACCCGTGGTGACTTTGAGAAAGCAATAGACAAGAAACGTCCGGTTCTTAGTGTGGCAGTGACCCGCTTCCCGTTCGTCCGTCTGTTACTCAAACGAATCAAGGAGGTGTTCTGATGGGACTGTTAGATTGTGAAGCCTGGGAAAAAGAAAACCCGCCAGTACAAGCAACTGGGTGTATAGCTTGCTTAGAGAAAGATTACCGTTATCCACACACCTGTAACAAAGGAGCTAACGATATGACCGAACGTG‑AACAAGAGATGATCATTAAGTTGATAGA                                                                                                                                                                                                                                                                                                                   >  1:729090/1‑301`  
  `ttataagagacagCATCATTATCCCCCGGGGTGACTTTGAGAAAGCAATAGACAAGAAACGTCCGGTTCTTAGTGTGGCAGTGACCCGCTTCCCGTTCGTCCGTCTGTTACTCAAACGAATCAAGGAGGTGTTCTGATGGGACTGTTAGATGGTGAAGCCTGGGAAAAAGAAAACCCGCCAGTACAAGCAACTGGGTGTATAGCTTGCTTAGAGAAAGATGACCGTTATCCACACACCTGTAACAAAGGAGCTAACGATATGACCGAACGTG‑AACAAGAGATGATCATTAAGTTGATAGAC                                                                                                                                                                                                                                                                                                                  <  1:269087/288‑1`  
  `gtataagagacagCATCATTATCACCCGTGGTGACTTTGAGAAAGCAATAGACAAGAACCGTTCGGTTCTTAGTGTGGCAGTGACCCGCTTCCCGTTCGTCCGTCTGTTACTCAAACGAATCAAGGAGGTGTTCTGATGGGACTGTTAGATGGTGAAGCCTGGGAAAAAGAAAACCCGCCAGTACAAGCAACTGGGTGTATAGCTTGCTTAGAGAAAGATGACCGTTATCCACACACCTGTAACAAAGGAGCTAACGATATGACCGAACGTG‑AACAAGAGATGATCATTAAGTTGATAGAC                                                                                                                                                                                                                                                                                                                  <  1:554458/288‑1`  
  `gtataagagacagCATCATTATCACCCGTGGTGACTTTGAGAAAGCAATAGACAAGAAACGTCCGGTTCTTAGTGTGGCAGTGACCCGCTTCCCGTTCGTCCGTCTGTTACTCAAACGAATCAAGGAGGTGTTCTGATGGGACTGTTAGATGGTGAAGCCTGGGAAAAAGAAACCCCGCCAGTACAAGCAACTGGGTGTATAGCTTGCTTAGAGAAAGATGACCGTTATCCACACACCTGTAACAAAGGAGCTAACGATATGACCGAACGTG‑AACAAGAGATGATCATTAAGTTGATAGAC                                                                                                                                                                                                                                                                                                                  <  1:101562/288‑1`  
  `gtataagagacagCATCATTATCACCCGTGGTGACTTTGAGAAAGCAATAGACAAGAAACGTCCGGTTCTTAGTGTGGCAGTGACCCGCTTCCCGTTCGTCCGTCTGTTACTCAAACGAATCAAGGAGGTGTTCTGATGGGACTGTTAGATGGTGAAGCCTGGGAAAAAGAAAACCCGCCAGTACAAGCAACTGGGTGTATAGCTTGCTTAGAGAAAGATGACCGTTATCCACACACCTGTAACAAAGGAGCTAACGATATGACCGAACGTG‑AACAAGAGATGATCATTAAGTTGATAGAC                                                                                                                                                                                                                                                                                                                  <  1:638159/288‑1`  
  `gACCGAGCGTCCACATCATTATCACCCGTGGTGACTTTTAGAAAGCAATAGACAAGAAACGTCCGGTTCTTAGTGTGGCAGTGACCCGCTTCCCGTTCGTCCGTCTGTTACTCAAACGAATCAAGGAGGTGTTCTGATGGGACTGTTAGATGGTGAATCCTGGGAAAAAGAAAACCCCCCAGTACAAGCAACTGGGTGTATAGCTTGCTTAGAGAAAGATGACCGTTATCCACACACCTGTAACAAAGGAGCTAACGATATGACCGAACGTG‑AACAAGAGATGATCATTAAGTTGATAGAC                                                                                                                                                                                                                                                                                                                  <  1:690068/300‑1`  
  `gACCGAGCGTCCAAATCATTATCACCCGTGGTGACTTTGAGAAAGCAATAGACAAGAAACGTCCGGTTCTTAGTGTGGCAGTGACCCGCTTCCCGTTCGTCCGTCTGTTACTCAAACGAATCAAGGAGGTGTTCTGATGGGACTGTTAGATGGTGAAGCCTGGGAAAAAGAAAACCCGCCAGTACAAGCAACTGGGTGTATAGCTTGCTTAGAGAAAGATGACCGTTATCCACACACCTGTAACAAAGGAGCTAACGATATGACCGAACGTG‑AACAAGAGATGATCATTAAGTTGATAGAC                                                                                                                                                                                                                                                                                                                  <  1:437342/300‑1`  
  `cgtgggttcggagatttgtataagagacaGGTGACTTTGAGAAAGCAATAGACAAGAAACGTCCGGTTCTTAGTGTGGCAGTTACCCGCTTCCCGTTCGTCCGTCTGTTACTCAAACGAATCAAGGAGGTGTTCTGATGGGACTGTTAGATGGTGAAGCCTGGGAAAAAGAAAACCCGCCAGTACAAGCAACTGGGTGTATAGCTTGCTTAGAGAAAGATGACCGTTATCCACACACCTGTAACAAAGGAGCTAACGATATGACCGAACGTG‑AACAAGAGATGATCATTAAGTTGATAGAC                                                                                                                                                                                                                                                                                                                  <  1:572144/272‑1`  
  `cacctggcggcaaaATCAGTAGCACCCGGGGTGACTTGGAGAAAGCAATAGACAAGAAAGTGCCGGTTCTTGGTGTGGCAGTGACCCGCGTCCCGTTCGTCCGGCTGTTACTCAAACGATGCAAGGAGTTGGTCGGAGGGGACTGTGAGATGGTGAAGCTTGGGAAAAAGAAAACCCGCCAGTACAAGCAACTGGGTGTATAGCTTGCTTAGAGAAAGATGACCGTTATCCACACACCTGTAACAAAGGAGCTAACGATATGACCGAACGTG‑AACAAGAGATGATCATTAAGTTGATAGAC                                                                                                                                                                                                                                                                                                                  <  1:435287/287‑1`  
  `atgtgtataagagacagATTATCACCCGTGGTTACTTTGAGAAAGCAATAGACAAGAAACGTCCTGTTCTTAGTGTGGCAGTAACCCGCTTCCCGTTCGTCCGTCTGTTACTCAAACGAATCAAGGAGGTGTTCTGATGGTACTGTTAGATGGTGAAGCCTGGGAAAAAGAAAACCCGCCAGTACAAGCAACTGGGTGTATAGCTTGCTTAGAGAAAGATGACCGTTATCCACACACCTGTAACAAAGGAGCTAACGATATGACCGAACGTG‑AACAAGAGATGATCATTAAGTTGATAGAC                                                                                                                                                                                                                                                                                                                  <  1:546248/284‑1`  
  `aACCGAGCGTCCACATCATTATCACCCGTGGTGACTTTGAGAAAGCAATAGACAAGAAACGTCCGGTTCTTAGTGTGGCAGTGACCCGCTTCCCGTTCGTCCGTCTGTTACTCAAACGAATCAAGTAGGTGTTCTGATGGGACTGTTAGATGGTGAAGCCTGGGAAAAAGAAAACCCGCCAGTACAAGCAACTGGGTGTATAGCTTGCTTAGAGAAAGATGACCGTTATCCACACACCTGTAACAAAGGAGCTAACGATATGACCGAACGTG‑AACAAGAGATGATCATTAAGTTGATAGAC                                                                                                                                                                                                                                                                                                                  <  1:104839/300‑1`  
  `aACCGAGCGTCCAAATCATTATAACCGTTGGTGACTTTGAGAAAGAAATAGACAAGAAACTGCGGGTTATTGTGGTGGCAGTGACCAGCTTCCCGGTCGTCCTTCTGGTACTTAAACGAATCAAGGAGGTGTTCTGATGGGACTGTTAGATGGTGAAGCCGGGGAAAAAGAAACCCCGCCAGTACAAGCAACTGGGTGTATAGCTTGCTTAGAGAAAGATGACCGTTATCCACACACCTGTAACAAAGGAGCTAACGATATGACCGAACGTG‑AACAAGAGATGATCATTAAGTTGATAGAC                                                                                                                                                                                                                                                                                                                  <  1:688101/300‑1`  
  `aACCGAGCGTCAAAATCATTATCACCCGTGGTGAGTTGGGAAAAGCAATAGACAAGAAACGTCCGGTTCTTAGTGTGGCAGTGACCCGCTTCCCGTTCGTCCGTCTGTTACTCAAACGAATCAAGGAGGTGTTCTGATGGGACTGTTAGATGGTGAAGCCTGGGAAAAAGAAAACCCGCCAGTACAAGCAACTGGGTGTATAGCTTGCTTAGAGAAAGATGACCGTTATCCACACACCTGTAACAAAGGAGCTAACGATATGACCGAACGTG‑AACAAGAGATGATCATTAAGTTGATAGAC                                                                                                                                                                                                                                                                                                                  <  1:750032/300‑1`  
  `CACCGAGCGTCCCCATCATAATCACCCGTGGTAACTATGAGAAAGCAATAGAGTAGAACCTGCAGGTACTTAGTGTGGCAGTGCCCCGTTTCCCGTTCGTCCGGCAGTTACTCAAACGAATCAAGGAGGTGTTCGGATGGAACTGTGAAATGGTGAAGCCTGGAAAAAAGAAAACCCGCCAGTACAAGCAACTGGGTGTATAGCTTGCTTAGAGAAAGATGACCGTTATCCACACACCTGTAACAAAGGAGCTAACGATATGACCGAACGTG‑AACAAGAGATGATCATTAAGTTGATAGAC                                                                                                                                                                                                                                                                                                                  <  1:780626/301‑1`  
  `CACCGAGCGTCCACATCATTATCACCCGTGGTGACTTTTAGAAAGCAATAGACAAGAAACGTCCGGTTCTTAGTGTGGCAGTGACCCGCTTCCCGTTCGTCCGTCTGTTACTCAAACGAATCAAGGAGGTGTTCTGATGGGACTGTTAGATGGTGAAGCCTGGGAAAAAGAAAACCCGCCAGTACAAGCAACTGGGTGTATAGCTTGCTTAGAGAAAGATGACCGTTATCCACACACCTGTAACAAAGGAGCTAACGATATGACCGAACGTG‑AACAAGAGATGATCATTAAGTTGATAGAC                                                                                                                                                                                                                                                                                                                  <  1:336604/301‑1`  
  `CACCGAGCGTCCACATCATTATCACCCGTGGTGACTTTGAGAAAGCAATAGACAAGAAACGTCCGGTTCTTAGTGTGGCAGTGACCCGCTTCCCGTTCGTCCGTCTGTTACTCAAACGAATCAAGGAGGTGTTCTGATGGGACTGTTAGATGGTGAAGCCTGGGAAAAAGAAAACCCGCCAGTACAAGCAACTGGGTGTATAGCTTGCTTAGAGAAAGATGACCGTTATCCACACACCTGTAACAAAGGAGCTAACGATATGACCGAACGTG‑CACAAGAGATGATCATTAAGTTGATAGAC                                                                                                                                                                                                                                                                                                                  <  1:306771/301‑1`  
  `CACCGAGCGTCCACATCATTATCACCCGTGGTGACTTTGAGAAAGCAATAGACAAGAAACGTCCGGTTCTTAGTGTGGCAGTGACCCGCTTCCCGTTCGTCCGTCTGTTACTCAAACGAATCAAGGAGGTGTTCTGATGGGACTGTTAGATGGTGAAGCCTGGGAAAAAGAAAACCCGCCAGTACAAGCAACTGGGTGTATAGCTTGCTTAGAGAAAGATGACCGTTATCCACACACCTGTAACAAAGGAGCTAACGATATGACCGAACGTG‑AACAAGAGATGATCATTAAGTTGATAGAC                                                                                                                                                                                                                                                                                                                  <  1:714966/301‑1`  
  `CACCGAGCGTCCACATCATTATCACCCGTGGTGACTTTGAGAAAGCAATAGACAAGAAACGTCCGGTTCTTAGTGTGGCAGTGACCCGCTTCCCGTTCGTCCGTCTGTTACTCAAACGAATCAAGGAGGTGTTCTGATGGGACTGTTAGATGGTGAAGCCTGGGAAAAAGAAAACCCGCCAGTACAAGCAACTGGGTGTATAGCTTGCTTAGAGAAAGATGACCGTTATCCACACACCTGTAACAAAGGAGCTAACGATATGACCGAACGTG‑AACAAGAGATGATCATTAAGTTGATAGAC                                                                                                                                                                                                                                                                                                                  <  1:502308/301‑1`  
  `CACCGAGCGTCCACATCATTATCACCCGTGGTGACATTGGGAAAGCAATAGACAAGAAACGTCCGGTTCTTAGTGTGGCAGTGACCCGCTTCCCGTTCGTCCGTCTGTTACTCAAACGAATCAAGGAGGTGTTCTGATGGGACTGTTAGATGGTGAAGCCTGGGAAAAAGAAAACCCGCCAGTACAAGCAACTGGGTGTATAGCTTGCTTAGAGAAAGATGACCGTTATCCACACACCTGTAACAAAGGAGCTAACGATATGACCGAACGTG‑AACAAGAGATGATCATTAAGTTGATAGAC                                                                                                                                                                                                                                                                                                                  <  1:465668/301‑1`  
  `CACCGAGAGTCCACATCATTATCACCCGTGGTGACTTTGAGAAAGCAATAGACAAGAAACGTCCGGTTCTTAGTGGGGCAGTGGCCCGCTTCCCGTTCGTCCGTCTGTTACTCAAACGAATCAAGGAGGTGTTCTGATGGGACTGTTAGATGGTGAAGCCTGGGAAAAAGAAAACCCGCCAGTACAAGCAACTGGGTGTATAGCTTGCTTAGAGAAAGATGACCGTTATCCACACACCTGTAACAAAGGAGCTAACGATATGACCGAACGTG‑AACAAGAGATGATCATTAAGTTGATAGAC                                                                                                                                                                                                                                                                                                                  <  1:578816/301‑1`  
    `agGAGCGTCCACATCATTATCACCCGTGGTGACTTGGATAAAGCAATAGACAAGAAACGTCCGGTTCTTAGTGTGGCAGTGACCCGCTTCCCGTTCGTCCGTCTGTTACTCAAACGAATCAAGGAGGTGTTCTGATGGGACTGTTAGATGGTGAAGCCTGGGAAAAAGAAAACCCGCCAGTACAAGCAACTGGGTGTATAGCTTGCTTAGAGAAAGATGACCGTTATCCACACACCTGTAACAAAGGAGCTAACGATATGACCGAACGTG‑AACAAGAGATGATCATTAAGTTGATAGACAA                                                                                                                                                                                                                                                                                                                <  1:258344/299‑1`  
    `CCGAGCGTCCACATCATTATCACCCGTGGTGACTTTGAGAAAGCAATAGACAAGAAACGTCCGGTTCTTAGTGTGGCAGTGACCCGCTTCCCGTTCGTCCATCTGTTACTCAAACGAATCAAGGAGGTGTTCTGATGGGACTGTTAGATGGTGAAGCCTGGGAAAAAGAAAACCCGCCAGTACAAGCAACTGGGTGTATAGCTTGCTTAGAGAAAGATGACCGTTATCCACACACCTGTAACAAAGGAGCTAACGATATGACCGAACGTG‑AACAAGAGATGATCATTAAGTTGATAGACAA                                                                                                                                                                                                                                                                                                                >  1:678661/1‑301`  
    `CCGAGCGTCAACATCATTATCACCCGTGGTGACTTTGAGAAAGCAATAGACAAGAAACGTCCGGTTCTTAGGGTGGCAGTGACCCGCTTCCCGTTCGTCCGTCTGTTACTCAAACGAATCAAGGAGGTGTTCTGATGGGACTGTTAGATGGTGAAGCCTGGGAAAAAGAAAACCCGCCAGTACAAGCAACTGGGTGTATAGCTTGCTTAGAGAAAGATGACCGTTATCCACACACCTGTAACAAAGGAGCTAACGATATGACCGAACGTG‑AACAAGAGATGATCATTAAGTTGATAGACAA                                                                                                                                                                                                                                                                                                                <  1:374630/301‑1`  
     `tgataagagACAGCATTATCACCGGTGGTGACTTTGAGAAAGCAATAGACAAGAAACGTCCGGTTCTTAGTGTGGAAGTGACCCGCTTCCCGTTCGTCCGTCTGTTACTCAAACGAATCAAGGAGGTGTTCTGATGGGACTGTTAGATGGTGAAGCCGGGGAAAAAGAAAACCCGCCAGTACAAGCAACTGGGTGTATAGCTTGCTTAGAGAAAGATGACCGTTATCCACACACCTGTAACAAAGGAGCTAACGATATGACCGAACGTG‑AACAAGAGATGATCATTAAGTTGATAGACAAT                                                                                                                                                                                                                                                                                                               <  1:731591/292‑1`  
      `GAGCGTCCACATCATTATCACCCGTGGTGACTTTGAGAAAGCAATAGACAAGAAACGTCCGGTTCTTAGTGTGGCAGTGACCCGCTTCCCGTTCGTCCGTCTGTTACTCGAACGAATCAAGGAGGTGTTCTGATGGGACTGTTAGATGGTGAAGCCTGGGAAAAAGAAAACCCGCCAGTACAAGCAACTGGGTGTATAGCTCGCTTAGAGAAAGATGACCGTTATCCACACACCTGTAACAAAGGAGCTAACGATATGACCGAACGTC‑AAAAATAGATGATCATTAAGTTGATTGCCAAca                                                                                                                                                                                                                                                                                                              >  1:126310/1‑299`  
      `GAGCGTCCACATCATTATCACCCGTGGTGACTTTGAGAAAGCAATAGACAAGAAACGTCCGGTTCTTAGTGTGGCAGTGACCCGCTTCCCGTTCGTCCGTCTGTTACTCAAACGAATCAAGGAGGTGTTCTGATGGGACTGTTAGATGGTGAAGCCTGGGAAAAAGAAAACCCGCCAGTACAAGCAACTGGGTGTATAGCTTGCTTAGAGAAAGATGACCGTTATCCACACACCTGTAACAAAGGAGCTAACGATATGACCGAACGTG‑AACAAGAGATGATCATTAAGTTGATAGACAATA                                                                                                                                                                                                                                                                                                              >  1:629870/1‑301`  
       `AGCGTCCACATCATTATCACCCGTGGTGACTTTGAGAAAGCAATAGACAAGAAACGTCCGGTTCTTAGTGTGGCAGTGACCCGCTTCCCGTTCGTCCGTCTGTTACTCAAACGAATCAAGGAGGTGTTCTGATGGGACTGTTAGATGGTGAAGCCTGGGAAAAAGAAAACCCGCCAGTACAAGCAACTGGGTGTATAGCTTGCTTAGAGAAAGATGACCGTTATCCACACACCTGTAACAAAGGAGCTAACGATATGACCGAACGTG‑AACAATAGATGATCATTAAGTTGATAGACAATAA                                                                                                                                                                                                                                                                                                             >  1:566585/1‑301`  
       `AGCGTCCACATCATTATCACCCGTGGTGACTTTGAGAAAGCAATAGACAAGAAACGTCCGGTTCTTAGTGTGGCAGTGACCCGCTTCCCGTTCGTCCGTCTGTTACTCAAACGAATCAAGGAGGTGTTCTGATGGGACTGTTAGATGGTGAAGCCTGGGAAAAAGAAAACCCGCCAGTACAAGCAACTGGGTGTATAGCTTGCTTAGAGAAAGATGACCGTTATCCACACACCTGTAACAAAGGAGCTAACGATATGACCGAACGTG‑AACAAGAGATGATCATTAAGTTGATAGACAATAA                                                                                                                                                                                                                                                                                                             <  1:642457/301‑1`  
         `CGTCCACATCATTATCACCCGTGGTGACTTTGAGAAAGCAATAGACAAGAAACGTCCGGTTCTTAGTGTGGCAGTGACCCGCTTCCCGTTCGTCCGTCTGTTACTCAAACGAATCAAGGAGGTGTTCTGATGGGACTGTTAGATGGTGAAGCCTGGGAAAAAGAAAACCCGCCAGTACAAGCAACTGGGTGTATAGCTTGCTTAGAGAAAGATGACCGTTATCCACACACCTGTAACAAAGGAGCTAACGATATGACCGAACGTG‑AACAAGAGATGATCATTAAGTTGATAGACctgtctc                                                                                                                                                                                                                                                                                                           >  1:36975/1‑294`  
         `CGTCCACATCATTATCACCCGTGGTGACTTTGAGAAAGCAATAGACAAGAAACGTCCGGTTCTTAGCGTGGCAGTGACCCGCTTCCCGTTCGTCCGTCTGTTACTCAAACGAATCAAGGAGGTGTTCTGATGGGACTGTTAGATGGTGAAGCCTGGGAAAAAGAAAACCCGCCAGTACAAGCAACTGGGTGTATAGCTTGCTTAGAGAAAGATGACCGTTATCCACACACCTGTAACAAAGGAGCTAACGATATGACCGAACGTG‑AACAAGAGATGATCATTAAGTTGATAGACAATActg                                                                                                                                                                                                                                                                                                           >  1:802668/1‑298`  
           `TCCACATCATTATCACCCGTGGTGACTTTGAGAAAGCTATAGACAAGAAACGTCCGGTTCTTAGTTTGGCATTGACCCGCTTCCCGTTCGTCCGTCTGTTACTCAAACGAATCCAGGAGGTGTTCTGATGGGCCTGTTAGATGGTTAAGCCTTGGACAACGAAAACCCGCCAGTACAAGCACCTGGGTGTATAGCTTGCTTAGAGAAAGATGACCGTTATCCACACACCTGTAACAAAGGAGCTAACGATATGACCGAACGTG‑AACAAGAGATGATCCTTACGTTGATAGACctgtccctt                                                                                                                                                                                                                                                                                                         >  1:810953/1‑292`  
           `TCCACATCATTATCACCCGTGGTGACTTTGAGAAAGCAATAGACAAGAAACGTCCGGTTCTTAGTGTGGCAGTGACCCGCTTCCCGTTCGTCCGTCTGTTACTCAAACGAATCAAGGAGGTGTTCTGATGGGACTGTTAGATGGTGAAGCCTGGGAAAAAGAAAACCCGCCAGTACAAGCAACTGGGTGTATAGCTTGCTTAGAGAAAGATGACCGTTATCCACACACCTGTAACAAAGGAGCTAACGATATGACCGAACGTG‑AACAAGAGATGATCATTATGTTGATAGACctgtctctt                                                                                                                                                                                                                                                                                                         >  1:512269/1‑292`  
              `ACATCATTCTCACCCGTGGTGACTTTGAGAAAGCAATAGACAAGAAACGTCCGGTTCTTAGTGTGGCAGTGACCCGCTTCCCGTTCGTCCGTCTGTTACTCAAACGAATCAAGGAGGTGTTCTGATGGGACTGTTAGATGGTGAAGCCTGGGAAAAAGAAAACCCGCCAGTACAAGCAACTGGGTGTATAGCTTGCTTAGAGAAAGATGACCGTTATCCACACACCTGTAACAAAGGAGCTAACGATATGACCGAACGTG‑AACAAGAGATGATCATTAAGTTGATATACctgtctcttata                                                                                                                                                                                                                                                                                                      >  1:187065/1‑289`  
               `CATCATTATCACCCGTGGTGACTTTGAGAAAGCAATAGACAAGAAACGTCCGGTTCTTAGTGTGGCAGTGACCCGCTTCCCGTTCGTCCGTCTGTTACTCAAACGAATCAAGGAGGTGTTATGATGGGACTGTTAGATGGTGAAGCCTGGGAAAAAGAAAACCCGCCAGTACAAGCAACTGGGTGTATAGCTTGCTTAGAGAAAGATGACCGTTATCCACACACCTGTAACAAAGGAGCTAACGATATGACCGAACGTG‑AACAAGAGATGATACTTAAGTTGATAGACAATAActgtctct                                                                                                                                                                                                                                                                                                     >  1:412319/1‑293`  
               `CATCATTATCACCCGTGGTGACTTTGAGAAAGCAATAGACAAGAAACGTCCGGTTCTTAGTGTGGCAGTGACCCGCTTCCC‑TTCGTCCGGCTGTTACTCAAACGAATCAAGAAAGTGTTCTGGTGGGACTGGTAGATGGTTAAACCTGGGGAAAAGAAAACCCGCCAGTAAAAACCACTGGGTGTATAGCTTGCTTAGAGAAAAATTAACGGTATCCACACACCTGTAACAAAGGAGCTAACGAAATTAACGAACGTGCAACAA‑AGATGAACATTAAGTTGATAGAactgtctcttaaaac                                                                                                                                                                                                                                                                                                    >  1:672963/1‑286`  
                 `TCATTATCACCCGTGGTGATTTTGAGAAAGCAATAGACAAGAAACGTCCGGTTCTTAGTGTTGCATTGACCCGCTTCCCGTTCGTCCGTCTGTTACTCAAACGAATCAAGGAGGTGTTCTGATGGGTCTGTTAGATGGTGAAGCCTGGGAAAAAGATAACCCGCCAGTACAAGCAACTGGTTTTATAGCTTGCTTAGATAAAGATTACCATTATCCACACAACTTTAACAAAGGAGCTCACTATATGACCTAACATG‑AACAAGATATGATCATTAAGTTTATATCCAATActgatgttcgc                                                                                                                                                                                                                                                                                                   >  1:527600/1‑290`  
                   `ATTATCACCCGTGGTGACTTTGAGAAAGCAATAGACAAGAAACGTCCGGTTCTTAGTGTGGCAGTGACCCGCTTCCCGTTCGTCCGTCTGTTACTCAAACGAATCAAGGAGGTGTTCTGATGGGACTGTTAGATGGTGAAGCCTGGGAAAAAGAAAACCCGCCAGTACAAGCAACTGGGTGTATAGCTTGCTTAGAGAAAGATGACCGTTATCCACACACCTGTAACAAAGGAGCTAACGATATGACCGAACGTG‑AACAAGAGATGATCATTAAGTTGATAGACctgtctcttatacacat                                                                                                                                                                                                                                                                                                 >  1:357099/1‑284`  
                           `CCGTGGTGACTTTGAGAAAGCAATAGACAAGAAACGTCCGGTTCTTAGTGTGGCAGTGACCCGCTTCCCGTTCGTCCGTCTGTTACTCAAACGAATCAAGGAGGTGTTCTGATGGGACTGTTAGATGGTGAAGCCTGGGAAAAAGAAAACCCGCCAGTACAAGCAACTGGGTGTATAGCTTGCTTAGAGAAAGATGACCGTTATCCACACACCTGTAACAAAGGAGCTAACGATATGACCGAACGTG‑AACAAGAGATGATCATTAAGTTGATAGACAATAAGAAGTTTTGGGCTACCGTAG                                                                                                                                                                                                                                                                                         <  1:520329/301‑1`  
                            `CGTGGTGACTTTGAGAAAGCAATAGACAAGAAACGTCCGGTTCTTAGTGTGGCAGTGACCCGCTTCCCGTTCGTCCGTCTGTTACTCAAACGAATCAAGGAGGTGTTCTGATTGGACTGTTAGATGGTGAAGCCTGGGCAAAAGAAAACCCGCCAGTACAAGCAACTGGGTGTATAGCTTGCTTAGAGAAAGATGACCGTTATCCACACACCTGTAACAAAGGAGCTAACGATATGACCGAACGTG‑AACAAGAGATGATCATTAAGTTGATAGACctgtctcttatacacatctccgagcc                                                                                                                                                                                                                                                                                        >  1:425746/1‑275`  
                                                    `GACAAGAAACGTCCGGTTCTTAGTGTGGCAGTGACCCGCTTCCCGTTCGTCCGTCTGTTACTCAAACGAATCAAGGAGGTGTTCTGATGGGACTGTTAGATGGTGAAGCCTGGGAAAAAGAAAACCCGCCAGTACAAACAACTGGGTGTATAGCTTGCTTAGAGAAAGATGACCGTTATCCACACACCTGTAACAAAGGAGCTAACGATATGACCGAACGTG‑AACAAGAGATGATCATTAAGTTGATAGACAATAAGAAGTTTTGGGCTACCGTAGAGTCCTCGGAGCATTCCTTCGAGGT                                                                                                                                                                                                                                                                >  1:347494/1‑301`  
                                                                                                                                       `CTGATGGGACTGTTAGATGGTGAAGCCTGGGAAAAAGAAAACCCGCCAGTACAAGCAACTGGGTGTATAGCTTGCTTAGAGAAAGATGACCGTTATCCACACACCTGTAACAAAGGAGCTAACGATATGACCGAACGTG‑AACAAGAGATGATCATTAAGTTGATAGACAATAAGAAGTTTTGGGCTACCGTAGAGTCCTCGGAGCATTCCTTCGAGGTTCCAATCTACGCTGAGACCCTAGACGAAGCTCTGGAGTTAGCCGAATGGCAATACGTTCCGGCTGGCTTTGAGGctgtctctt                                                                                                                                                                             >  1:770225/1‑292`  
                                                                                                                                              `agagacagGATGGTGAAGCCTGGGAAAAAGAAAACCCGCCAGTACAAGCAACTGGGTGTATAGATTGCTTAGAGAAAGATGACCGTTATCCACACACCTGTAACAAAGGAGCTAACGATATGACCGAACGTG‑AACAAGAGATGATCATTAAGTTGATAGACAATAAGAAGTTTTGGGCTACCGTAGAGTCCTCGGAGCATTCCTTCGAGGTTCCAATCTACGCTGAGACCCTAGACGAAGCTCTGGAGTTAGCCGAATGGCAATACGTTCCGGCTGGCTTTGAGGTTACTCGTGTGCGTCC                                                                                                                                                                      <  1:605045/293‑1`  
                                                                                                                                                    `TAGATGTTGAAGCCTGGGAAAAAGAACACCCGCCAGTACAAGCAACTGGGTGTATAGCTTGCTTAGAGAAAGATGACCGTTATCCACACACCTGTAACAAAGGAGCTAACGATATGACCGAACGTG‑AACAAGAGATGATCATTAAGTTTATAGACAATAAGAAGTTTTGGGCTACCGTAGAGTCCTCGTAGCATTCCTTCGAGGTTCCAATCTACGCTGAGACCCTAGACGAAGCTCTGGAGTTAGCCGAATGGCAATACGTTCCGGCTGGCTTTGAGGTTACTCGTGTGCGTCCTTGTGT                                                                                                                                                                <  1:87360/301‑1`  
                                                                                                                                                                                   `GCCAGTACAAGCAACTGGGTGTATAGCTTGCTTAGAGAAAGAGGACCGTTATCCAAACACCTGTAACAAAGGAGCTAACGATATGACCGAACGTG‑AACAAGAGATGATCATTAAGTTGATAGACAATAAGAAGTTTTGGGCTACCGTAGAGTCCTCGGAGCATTCCTTCGAGGTTCCAATCTACGCTGAGACCCTAGACGAAGCTCTGGAGTTAGCTGAATGGCAATACGTTCCGGCTGGCTTTGAGGTTACTCGTGTGCTTCCTTGTGTAGCACCGAAGTAATACGACTCACTATTAGGG                                                                                                                                 <  1:757321/301‑1`  
                                                                                                                                                                                                     `GTGTATAGCTTGCTTAGAGAAAGATGACCGTTATCCACACACCTGTAACAAAGGAGCTAACGATATTACCGAACGTG‑AACAAGAGATGATCATTAAGTTGATAGACAATAAGAAGTTTTGGGCTACCGTAGAGTCCTCGGAGCATTCCTTCGAGGTTCCAATCTACGCTGAGACCCTAGACGAAGCTCTGGAGTTAGCCGAATGGCAATACGTTCCGGCTGGCTTTGAGGTTACTCGTGTGCGTCCTTGTGTAGCACCGAAGTAATACGACTCACTATTAGGGAAGACTCCCTCTGAGAAA                                                                                                               >  1:390546/1‑301`  
                                                                                                                                                                                                     `GTGTATAGCTTGCTTAGAGAAAGATGACCGTTATCCACACACCTGTAACAAAGGAGCTAACGATATGACCGAACGTG‑AACAAGAGATGATCATTAAGTTGATAGACAATAAGAAGTTTTGGGCTACCGTAGAGTCCTCGGAGCATTCCTTCGAGGTTCCAATCTACGCTGAGACCCTAGACGAAGCTCTGGAGTTAGCCGAATGGCAATACGTTCCGGCTGGCTTTGAGGTTACTCGTGTGCGTTCTTGTGTAGCACCGCAGTAATACGACTCACTATTAGGGctgtctcttatacacatc                                                                                                               >  1:524928/1‑283`  
                                                                                                                                                                                                           `AGCTTGCTTAGAGAAAGATGACCGTTATCCTCACACCTGTAACAAAGGAGCTAACGATATGACCGAACGTG‑AACAAGAGATGATCATTAAGTTGATAGACAATAAGAAGTTTTGGGCTACCGTAGAGTCCTCGGAGCATTCCTTCGAGGTTCCAATCTACGCTGAGACCCTAGACGAAGCTCTGGAGTTAGCCGAATGGCAATACGTTCCGGCTGGCTTTGAGGTTACTTGTGTGCGTCCTTGTTGAGCACCGAAGTAATACGACTCACTATTAGGGAAGACTCCCTCTGAGAAACCAAAC                                                                                                         >  1:61649/1‑301`  
                                                                                                                                                                                                                                                                   `ATATGACCGAACGTG‑AACAAGAGATGATCATTAAGTTGATAGACAATAAGAAGTGTTGGGCTACCGTAGAGTCCTCGGAGCATTCCTTCGAGGTTCCAATCTACGCTGAGACCCTAGACGAAGCTCTGGAGTTAGCCGAATGGCAATACGTTCCGGCTGGCTTTGAGGTTACTCGTGTGCGTCCTTGTGTAGCACCGAAGTAATACGACTCACTATTAGGGAAGACTCCCTCTGAGAAACCAAACGAAACCTAAAGGAGATTAACATTATGGCTAAGAAGATTTTCACCTCTGCGCTGGGT                                                 <  1:326260/301‑1`  
                                                                                                                                                                                                                                                                                    `gggaaatccaggttcacttgacagtgaaAATAAAAAGTTTTGGGCTACC‑TAAAGTCCTAGGAGCATACCTTCGAGGGTCCAATCTACGCTGGGACGATAGACGAAGGTGTGGAGTTAGCCAAATGGCAATACGTTCCGGCTGGCTTTGCGGTTACTCGTGGGCCTCCTTGTGTAGCACCGAAGTAATACGACTCAATATTAGCGAAGACTCCCTCGGAGAAACCAATCGAAACCTAAAGGAGATTAACATTATGGCTAAGAAGATTTTCACCTCTGCGCTGGGTACCGCTGAACCTTACGC                                <  1:389778/273‑1`  
                                                                                                                                                                                                                                                                                     `ggggctcggagatgtgtataagagacagATAAGAAGTTTTGGGCTACCGTAGAGTCCTCGGAGCATTCCTTCGAGGTTCCAATCTACGCTGAGACACTAGACGAAGCTCTGGAGTTAGCCGAATGGCAATACGTTCCGGCTGGCTTTGAGGTTACTCGTGTGCGTCCTTGTGTAGCACCGAAGTAATACGACTCACTATTAGGGAAGACTCCCTCTGAGAAACCAAACGAAACCTAAAGGAGATTAACATTATGGCTAAGAAGATTTTCACCTCTGCGCTGGTTACCGCTGAACCTTACGC                                <  1:223867/273‑1`  
                                                                                                                                                                                                                                                                                                   `TAAGTTGATAGACAATAAGAAGTTTTGGGCTACCGTAGAGTCCTCGGAGCATTCCTTCGAGGTTCCAATCTACGCTGAGACCCTAGACGAAGCTCTGGAGTTAGCCGAATGGCAATACGTTCCGGCTGGCTTTGAGGTTACTCGTGTGCGTCCTTGTGTAGCACCGAAGTAATACGACTCACTATTAGGGAAGACTCCCTCTGAGAAACCAAACGAAACCTAAAGGAGATTAACATTATGGCTAAGAAGATTTTCACCTCTGCGCTGGGTACCGCTGAACCTTACGCTTACATCGCCAAGC                  <  1:399598/301‑1`  
                                                                                                                                                                                                                                                                                                      `aagagacagCAATAAGAAGTTTTGGGCTACCGTAGAGTCCTCGGAGCATTCCTTCGAGGTTCCAATCTACGCTGAGACCCTAGACGAAGCTCTGGAGTTAGCCGAATGGCAATACGTTCCGGCTGGCTTTGAGGTTACTCGTGTGCGTCCTTGTGTAGCACCGAAGTAATACGACTCACTATTAGGGAAGACTCCCTCTGAGAAACCAAACGAAACCTAAAGGAGATTAACATTATGGCTAAGAAGATTTTCACCTCTTCGCTGGGTACCGCTGAACCTTACGCTTACATCGCCAAGCCGG               <  1:704648/292‑1`  
                                                                                                                                                                                                                                                                                                      `GTTGATAGACAATAAGAAGTTTTGGGCTACCGTAGAGTCATCGGAGCATTCCTTAGAGGTTCCAATCTACGCTGAGACCCTAGACGAAGCTCTGGAGTTAGCCGAATGGCAATACGTTCCGGCTGGCTTTGAGGTTACTCGTGTGCGTCCTTGTGTAGCAACGAAGTAATACGACTCACTATTAGGGAAGACTCCCTCTGAGAAACCAAACGAAACCTAAAGGAGATTAACATTATGGCTAAGAAGATTTTCACCTCTGCGCTGGGTACCGCTGAACCTTACGCTTACATCTCCAAGACGG               >  1:452363/1‑301`  
                                                                                                                                                                                                                                                                                                             `GACAATAAGAAGTTTTGGGCTACCGTAGAGTCCTCGGAGCATTCCTTCGAGGTTCCAATCTACGCTGATACCCGAGACGAAGCTCTGGAGTTAGCCGAATGGCAATACGTTCCGGGTGGCTTTGAGGTTATTCGTGTGCGTCCTTGTGTAGCACAGAAGTAATACGACTCACTATTAGGGAAGAATCCCTCTGAGAAACCAAACGAAACCTAAAGGAGATTAACATTATGGCTAAGAAGATTTTCACCTCTGCGCTGGGTACCGCTGAACCTTACGCTTACATCGCCAAGCCGGACTACGG        <  1:483057/301‑1`  
                                                                                                                                                                                                                                                                                                             `GACAATAAGAAGTTTTGGGCTACCGTAGAGTCCTCGGAGCATTCCTTCGAGGTTCCAATCTACGCTGAGACCCTAGACGAAGCTCTGGAGTTAGCCGAATGGCAATACGTTCCGGCTGGCTTTGAGGTTACTCGTGTGCGTCCTTGTGTATCACCGAAGTAATACGACTCACTATTAGGGAAGACTCCCTCTGAGAAACCAAACGAAACCTAAAGGAGATTAACATTATGGCTAAGAAGATTTTCACCTCTGCGCTGGGTACCGCTGAACCTTACGCTTACATCGCCAAGCCGGACTACGG        >  1:169769/1‑301`  
                                                                                                                                                                                                                                                                                                             `GACAATAAGAAGTTTTGGGCTACCGTAGAGTCCTCGGAGCATTCCTTCGAGGTTCCAATCTACGCTGAGACCCTAGACGAAGCTCTGGAGTTAGCCGAATGGCAATACGTTCCGGCTGGCTTTGAGGTTACTCGTGTGCGTCCTTGTGTAGCACCGAAGTAATACGACTCACTATTAGGGAAGACTCCCTCTGAGAAACCAAACGAAACCTAAAGGAGATTAACATTATGGCTAAGAAGATTTTCACCTCTGCGCTTGGTACCGCTGAACCTTACGCTTACATCGCCAAGCCGGACTACGG        <  1:617619/301‑1`  
                                                                                                                                                                                                                                                                                                             `GACAATAAGAAGTTTTGGGCTACCGTAGAGTCCTCGGAGCATTCCTTCGAGGTTCCAATCTACGCTGAGACCCTAGACGAAGCTCTGGAGTTAGCCGAATGGCAATACGTTCCGGCTGGCTTTGAGGTTACTCGTGTGCGTCCTTGTGTAGCACCGAAGTAATACGACTCACTATTAGGGAAGACTCCCTCTGAGAAACCAAACGAAACCTAAAGGAGATTAACATTATGGCTAAGAAGATTTTCACCTCTGCGCTGGGTACCGCTGAACCTTACGCTTACATCGCCAAGCCGGACTACGG        >  1:653413/1‑301`  
                                                                                                                                                                                                                                                                                                             `GACAATAAGAAGTTTTGGGCTACCGTAGAGTCCTCGGAGCATTCCTTCGAGGTTCCAATCTACGCTGAGACCCTAGACGAAGCTCTGGAGTTAGCCGAATGGCAATACGTTCCGGCTGGCTTTGAGGTTACTCGTGTGCGTCCTTGTGTAGCACCGAAGTAATACGACTCACTATTAGGGAAGACTCCCTCTGAGAAACCAAACGAAACCTAAAGGAGATTAACATTATGGCTAAGAAGATTTTCACCTCTGCGCTGGGTACCGCTGAACCTTACGCTTACATCGCCAAGCCGGACTACGG        <  1:37878/301‑1`  
                                                                                                                                                                                                                                                                                                              `cagATAAGAAGTTTTGGGCTACCGTAGAGTCCTCGGAGCATTCCTTCGAGGTTCCAATCTACGCTGAGACCCTAGACGAAGCTCTGGAGTTAGCCGAATGGAAATACGTTCCGGCTGGCTTTGAGGTTACTCGTGTGCGTCCTTGTGTAGCACCGAAGTAATACGACTCACTATTAGGGAAGACTCCCTCTGAGAAACCAAACGAAACCTAAAGGAGATTAACATTATGGCTAAGAAGATTTTCACCTCTGCGCTGGGTACCGCTGAACCTTACGCTTACATCGCCAAGCCGGACTACGGC       <  1:591988/298‑1`  
                                                                                                                                                                                                                                                                                                              `agAATAAGAAGTTTTGGGCTACCGTAGAGTCCTCGGAGCATTCCTTCGAGGTTCCAATCTACGCTGAGACCCTAGACGAAGCTCTGGAGTTAGCCGAATGGCAATACGTTCCGGCTGGCTTTGAGGTTACTCGTGTGCGTCCTTGTGTAGCACCGAAGTAATACGACTCACTATTAGGGAAGACTCCCTCTGAGAAACCAAACGAAACCTAAAGGAGATTAACATTATGGCTAAGAAGATTTTCACCTCTGCGCTGGGTACCGCTGAACCTTACGCTTACATCGCCAAGCCGGACTACGGC       <  1:47469/299‑1`  
                                                                                                                                                                                                                                                                                                              `aaAATAAGAAGTTTGGGGCTACCGTAGAGTCCTCGGAGCATTCCTTCGAGGTTCCAATCTACGCTGAGACCCTAGACGAAGCTCTGGAGTTAGCCGAATGGCAATACGTTCCGGCTGGCTTTGAGGTTACTCGTGTGCGTCCTTGTGTAGCACCGAAGTAATACGACTCACTATTAGGGAAGACTCCCTCTGAGAAACCAAACGAAACCTAAAGGAGATTAACATTATGGCTAAGAAGATTTTCACCTCTGCGCTGGGTACCGCTGAACCTTACGCTTACATCGCCAAGCCGGACTACGGC       <  1:28707/299‑1`  
                                                                                                                                                                                                                                                                                                              `ACAATTATAAGTTTTGGGCTACCCTAGAGTCCTCGGAACATTCCTTCGAGATTCCAATCTCCGCTGATACCCTAGACGCAGCTCTGGAGTTAGCCGAATGGCCATACGTTCCGGATGGCTTTGAGGTTACTCGTGTGCGTCCTTGTGTAGCACCGAAGTAATACGACTCACTATTAGGGAAGACTCCCTCTGAGAAGCCAAACGAAACCTAAAGGAGATTAACATTATGGCTAAGAAGATTTTCACCTCTGCGCTGGGTACCGCTGAACCTTACGCTTACATCGCCAAGCCGGACTACGGC       <  1:521719/301‑1`  
                                                                                                                                                                                                                                                                                                              `ACAATAAGAAGTTTTGGGCTACCGTAGAGTCCTCGGAGCATTCCTTCGAGGTTCCAATCTACGCTGAGACCCTAGACGAAGCTCTGGAGTTAGCCGAATGGCAATACGTTCCGGCTGGCTTTGAGGTTACTCGTGTGCGTCCTTGTGTAGCACCGAAGTAATACGACTCACTATTAGGGAAGACTCCCTCTGAGAAACCAAACGAAACCTAAAGGAGATTAACATTATGGCTAAGAAGATTTTCACCTCTGCGCTGGGTACCGCTGAACCTTACGCTTACATCGCCAAGCCGGACTACGGC       <  1:151523/301‑1`  
                                                                                                                                                                                                                                                                                                              `ACAATAAGAAGTTTTGGGCTACCGTAGAGTCCTCGGAGCATTCCTTCGAGGTTCCAATCTACGCTGAGACCCTAGACGAAGCTCTGGAGTTAGCCGAATGGCAATACGTTCCGGCTGGCTTTGAGGTTACTCGTGTGCGTCCTTGTGTAGCACCGAAGTAATACGACTCACTATTAGGGAAGACTCCCTCTGAGAAACCAAACGAAACCTAAAGGAGATTAACATTATGGCTAAGAAGATTTTCACCTCTGCGCTGGGTACCGCTGAACCTTACGCTTACATCGCCAAGCCGGACTACGGC       <  1:488500/301‑1`  
                                                                                                                                                                                                                                                                                                              `ACAATAAGAAGTTTTGGGCTACCGTAGAGTCCTCGGAGCATTCCTTCGAGGTTCCAATCTACGCTGAGACCCTAGACGAAGCTCTGGAGTTAGCCGAATGGCAATACGTTCCGGCTGGCTTTGAGGTTACTCGTGTGCGTCCTTGTGTAGCACCGAAGTAATACGACTCACTATTAGGGAAGACTCCCTCTGAGAAACCAAACGAAACCTAAAGGAGATTAACATTATGGCTAAGAAGATTTTCACCTCTGCGCTGGGTACCGATGAACCTTACGCTTACATCGCCAAGCCGGACTACGGC       <  1:66817/301‑1`  
                                                                                                                                                                                                                                                                                                              `ACAATAAGAAGTTTTGGGCTACCGTAGAGTCCTCGGAGCATTCCTTCGAGGTTCCAATCTACGCTGAGACCCTAGACGAAGCTCTGGAGTTAGCCGAATGGCAATACGTTCCGGCTGGCTTTGAGGTTACTCGTGTGCGTCCTTGTGTAGCACCGAAGTAATACGACGCACTATTAGGGAAGACTCCCTCTGAGAAACCAAACGAAACCTAAAGGAGATTAACATTATGGCTAAGAAGATTTTCACCTCTGCGCTGGGTACCGCTGAACCTTACGCTTACATCGCCAAGCCGGACTACGGC       <  1:493230/301‑1`  
                                                                                                                                                                                                                                                                                                              `ACAATAAGAAGTTTTGGGCTACCGTAGAGTCCTCGGAGCATTCCTTCGAGGTTCCAATCTACGCTGAGACCCTAGACGAAGCTCTGGAGTTAGCCGAATGGAAATACGTTCCGGCTGGCTTTGAGGTTACTCGTGTGCGTCCTTGTGTAGCACCGAAGTAATACGACTCACTATTAGGGAAGACTCCCTCTGAGAAACCAAACGAAACCTAAAGGAGATTAACATTATGGCTAAGAAGATTTTCACCTCTGCGCTGGGTACCGCTGAACCTTACGCTTACATCGCCAAGCCGGACTACGGC       <  1:118066/301‑1`  
                                                                                                                                                                                                                                                                                                              `ACAATAAGAAGTTTTGGGCTACCGTAGAGTCCTCGGAGCATTCCTTCGAGGTTCCAATCTACGCTGAGACCCCAGACGAAGCTCTGGAGTTAGCCGAATGGCAATACGTTCCGGCTGGCTTTGAGGTTACTCGTGTGCGTCCTTGTGTAGCACCGAAGTAATACGACTCACTATTAGGGAAGACTCCCTCTGAGAAACCAAACGAAACCTAAAGGAGATTAACATTATGGCTAAGAAGATTTTCACCTCTGCGCTGGGTACCGCTGAACCTTACGCTTACATCGCCAAGCCGGACTACGGC       <  1:35324/301‑1`  
                                                                                                                                                                                                                                                                                                              `ACAATAAGAAGTTTTGGGCTACCGTAGAGTCCTCGGAGCATTCCTTCGAGGTTCCAACCTACGCTGAGACCCTAGACGAAGCTCTGGAGTTAGCCGAATGGCAATACGTTCCGGCTGGCTTTGAGGTTACTCGTGTGCGTCCTTGTGTAGCACCGAAGTAATACGACTCACTATTAGGGAAGACTCCCTCTGAGAAACCAAACGAAACCTAAAGGAGATTAACATTATGGCTAAGAAGATTTTCACCTCTGCGCTGGGTACCGCTGAACCTTACGCTTACATCGCCAAGCCGGACTACGGC       <  1:537479/301‑1`  
                                                                                                                                                                                                                                                                                                              `ACAATAAGAAGTTTTGGGCTACCGTAGAGTCCTCGAAGCATTCCTTCGAGGTTCCAATCTACGCTGAGACCCTAGACGAAGCTCTGGAGTTAGCCGAATGGCAATACGTTCCGGCTGGCTTTGAGGTTACTCGTGTGCGTCCTTGTGTAGCACCGAAGTAATACGACTCACTATTAGGGAAGACTCCCTCTGAGAAACCAAACGAAACCTAAAGGAGATTAACATTATGGCTAAGAAGATTTTCACCTCTGCGCTGGGTACCGCTGAACCTTACGCTTACATCGCCAAGCCGGACTACGGC       <  1:205538/301‑1`  
                                                                                                                                                                                                                                                                                                               `CAATAAGACGTTTTGGGCTACCGTAGAGTCCTCGGAGCATTCCTTCGAGGTTCCAATCTACGCTGAGACCCTAGACGAATCTCTGGAGTTAGCCGAATGGCAATACGTTCCGGCTGGCTTTGAGGTTACTCGTGTGCGTCCTTGTGTAGCACCGAAGTAATACGACTCACTATTAGGGAAGACTCCCTCTGAGAAACCAAACGAAACCTAAAGGAGATTAACATTATGGCTAAGAAGATTTTCACCTCTGCGCTGGTTACCGCTGAACCTTACGCTTACATCGCCAAGCCGGACTACGGCA      >  1:722754/1‑301`  
                                                                                                                                                                                                                                                                                                               `CAATAAGAAGTTTTGGGCTACCGTAGAGTCCTCGGAGCATTCCTTCGAGGTTCCAATCTACGCTGAGACCCTAGACGAAGCTCTGGAGTTAGCCGAATGGCAATACGTTCCGGCTGGCTTTGAGGTTACTCGTGTGCGTCCTTGTGTAGCACCGAAGTAATACGACTCACTATTAGGGAAGACTCCCTCTGAGAAACCAAACGAAACCTAAAGGAGATTAACATTATGGCTAAGAAGATTTTCACCTCTGCGCTGGGTACCGCTGAACCTTACGCTTACATCGCCAAGCCGGCCTACGGCA      >  1:382368/1‑301`  
                                                                                                                                                                                                                                                                                                               `CAATAAGAAGTTTTGGGCTACCGTAGAGTCCTCGGAGCATTCCTTCGAGGTTCCAATCTACGCTGAGACCCTAGACGAAGCTCTGGAGTTAGCCGAATGGCAATACGTTCCGGCTGGCTTTGAGGTTACTCGTGTGCGTCCTTGTGTAGCACCGAAGTAATACGACTCACTATTAGGGAAGACTCCCTCTGAGAAACCAAACGAAACCTAAAGGAGATTAACATTATGGCTAAGAAGATTTTCACCTCTGCGCTGGGTACCGCTGAACCTTACGCTTACATCGCCAAGCCGGACTACGGCA      >  1:708577/1‑301`  
                                                                                                                                                                                                                                                                                                               `CAATAAGAAGTTTTGGGCTACCGTAGAGTCCTCGGAGCATTCCTTCGAGGTTCCAATCTACGCTGAGACCCTAGACGAAGCTCTGGAGTTAGCCGAATGGCAATACGTTCCGGCTGGCTTTGAGGTTACTCGTGTGCGTCCTTGTGTAGCACCGAAGTAATACGACTCACTATTAGGGAAGACTCCCTCTGAGAAACCAAACGAAACCTAAAGGAGATTAACATTATGGCTAAGAAGATTTTCACCTCTGCGCTGGGTACCGCTGAACCTTACGCTTACATCGCCAAGCCGGACTACGGCA      >  1:784947/1‑301`  
                                                                                                                                                                                                                                                                                                                `AATAAGAAGTTTTGGGCTACCGTAGAGTCCTCGGAGCATTTCTTCGAGGTTCCAATCTACGCTGAGACCCTAGACGAAGCTCTGGAGTTAGCCGAATGGCAATACGTTCCGGCTGGCTTTGAGGTTACTCGTGTGCGTCCTTGTGTAGCACCGAAGTAATACGACTCACTATTAGGGAAGACTCCCTCTGAGAAACCAAACGAAACCTAAAGGAGATTAACATTATGGCTAATAAGATTTTCACCTCTGCGCTGGGTACCGCTGAACCTTACGCTTACATCGCCAAGCCGGACTACGGCAA     <  1:419914/301‑1`  
                                                                                                                                                                                                                                                                                                                `AATAAGAAGTTTTGGGCTACCGTAGAGTCCTCGGAGCATTCCTTCGAGGTTCCAATCTACGCTGAGACCCTAGACGAAGCCCTGGAGTTAGCCGAATGGCAATACGTTCCGGCTGGCTTTGAGGTTACTCGTGTGCGTCCTTGTGTAGCACCGAAGTAATACGACTCACTATTAGGGAAGACTCCCTCTGAGAAACCAAACGAAACCTAAAGGAGATTAACATTATGGCTAAGAAGATTTTCACCTCTGCGCTGGGTACCGCTGAACCTTACGCTTACATCGCCAAGCCGGACTACGGCAA     <  1:323920/301‑1`  
                                                                                                                                                                                                                                                                                                                `AATAAGAAGTTTTGGGCTACCGTAGAGTCCTCGGAGCATTCCTTCGAGGTTCCAATCTACGATGAGACCCTAGACGAAGCTCTGGAGTTAGCCGAATGGCAATACGTTCCGGCTGGCTTTGAGGTTACTCGTGTGCGTCCTTGTGTAGCACCGAAGTAATACGACTCACTATTAGGGAAGACTCCCTCTGAGAAACCAAACGAAACCTAAAGGAGATTAACATTATGGCTAAGAAGATTTTCACCTCTGCGCTGGGTACCGCTGAACCTTACGCTTACATCGCCAAGCCGGACTACGGCAA     <  1:76554/301‑1`  
                                                                                                                                                                                                                                                                                                                `AATAAGAAGTTTTGGGATACCGTAGAGTCCTCGGAGCATTCCTTCGAGGTTCCAATCTACGCTGAGACCCTAGACGAAGCTCTGGAGTTAGCCGAATGGCAATACGTTCCGGCTGGCTTTGAGGTTACTCGTGTGCGTCCTTGTGTAGCACCGAAGTAATACGACTCACTATTAGGGAAGACTCCCTCTGAGAAACCAAACGAAACCTAAAGGAGATTAACATTATGGCTAAGAAGATTTTCACCTCTGCGCTGGGTACCGCTGAACCTTACGCTTACATCGCCAAGCCGGACTACGGCtg     >  1:40641/1‑299`  
                                                                                                                                                                                                                                                                                                                 `ATACGAAGTTTTGGGCTACCGTAGAGTCCTCGGAGCATTCCTTCGAGGTTCCAATCTACGCTGAGACCCTAGACGAAGCTCTGGAGTTAGCCGAATGGCAATACGTTCCGGCTGGCTTTGAGGTTACTCGTGTGCGTCCTTGTGTAGCACCGAAGTAATACGACTCACTATTAGGGAAGACTCCCTCTGAGAAACCAAACGAAACCTAAAGGAGATTAACATTATGGCTAAGAAGATTTTCACCTCTGCGCTGGGTACCGCTGAACCTTACGCTTACATCGCCAAGCCGGACctgtctctt    >  1:289931/1‑292`  
                                                                                                                                                                                                                                                                                                                 `ATAAGAAGTTTTGGGCTACCGTAGAGTCCTCGGAGCATTCCTTCGAGGTTCCAATCTACGCTGAGACCCTAGACGAAGCTCTGGAGTTAGCCGAATGGCAATACGTTCCGGCTGGCTTTGAGGTTACTCGTGTGCGTCCTTGTGTAGCACCGAAGTAATACGACTCACTATTAGGGAAGACTCCCTCTGAGAAACCAAACGAAACCTAAAGGAGATTAACATTATGGCTAAGAAGATTTTCACCTCTGCGCTGGGTACCGCTGAACCTTACGCTTACATCGCCATGCCGGACTACGGCAAC    >  1:472537/1‑301`  
                                                                                                                                                                                                                                                                                                                 `ATAAGAAGTTTTGGGCTACCGTAGAGTCCTCGGAGCATTCCTTCGAGGTTCCAATCTACGCTGAGACCCTAGACGAAGCTCTGGAGTTAGCCGAATGGCAATACGTTCCGGCTGGCTTTGAGGTTACTCGTGTGCGTCCTTGTGTAGCACCGAAGTAATACGACTCACTATTAGGGAAGACTCCCTCTGAGAAACCAAACGAAACCTAAAGGAGATTAACATTATGGCTAAGAAGATTTTCACCTCTGCGCTGGGTACCGCTGAACCTTACGCTTACATCGCCAAGCCGGACTACGGCAAC    >  1:480868/1‑301`  
                                                                                                                                                                                                                                                                                                                 `ATAAGAAGTTTTGGGCTACCGTAGAGTCCTCGGAGCATTCCTTCGAGGTTCCAATCTACGCTGAGACCCTAGACGAAGCTCTGGAGTTAGCCGAATGGCAATACGTTCCGGCTGGCTTTGAGGTTACTCGTGTGCGTCCTTGTGTAGCACCGAAGTAATACGACTCACTATTAGGGAAGACTCCCTCTGAGAAACCAAACGAAACCTAAAGGAGATTAACATTATGGCTAAGAAGATTTTCACCTCTGCGCTGGGTACCGCTGAACCTTACGATTACATCGCCAAGCCGGACTAAGGCAAC    >  1:274203/1‑301`  
                                                                                                                                                                                                                                                                                                                 `ATAAGAAGTTTTGGGCTACCGTAGAGTCCTCGGAGCATTCCTTCGAGGTTCCAATCTACGCTGAGACCCTAGACGAAGCTCTGGAGTTAGCCGAATGGCAATACGTTCCGGCTAGCTTTGAGGTTACTCGTGTGCGTCCTTGTGTAGCACCGAAGTAATACGACTCACTATTAGGGAAGACTCCCTCTGAGAAACCAAACGAAACCTAAAGGAGATTAACATTATGGCTAAGAAGATTTTCACCTCTGCGCTGGGTACCGCTGAACCTTACGCTTACATCGCCAAGCCGGACTACGGCAAC    >  1:241935/1‑301`  
                                                                                                                                                                                                                                                                                                                  `TAAGAAGTTTTGGGCTACCGTAGAGTCCTCGGAGCATTCCTTCGAGGTTCCAATCTACGCTGAGACCCTAGACGAAGCTCTGGAGTTAGCCGAATGGCAATACGTTCTGGCTGGCTTTGAGGTTACTCGTGTGCGTCCTTGTGTAGCACCGAAGTAATACGACTCACTATTAGGGAAGACTCCCTCTGAGAAACCAAACGAAACCTAAAGGAGATTAACATTATGGCTAAGAAGATTTTCACCTCTGCGCTGGGTACCGCTGAACCTTACGCTTACATCGCCAAGCCGGACTctgtctctt   >  1:734410/1‑292`  
                                                                                                                                                                                                                                                                                                                  `TAAGAAGTTTTGGGCTACCGTAGAGTCCTCGGAGCATTCCTTCGAGGTTCCAATCTACGCTGAGACCCTAGACGAAGCTCTGGAGTTAGCCGAATGGCAATACGTTCCGGCTGGCTTTGAGGTTACTCGTGTGCGTCCTTGTGTAGCACCGAAGTAATACGACTCACTATTAGGGAAGACTCCCTCTGAGAAACCAAACGAAACCTAAAGGAGATTAACATTATGGCTAAGAAGATTTTCACCTCTGCGCTGGGTACCGCTGAACCTTACGCTTACATCGCCAAGCCGGACTACGGCAACG   >  1:235613/1‑301`  
                                                                                                                                                                                                                                                                                                                  `TAAGAAGTTTTGGGCTACCGTAGAGTCCTCGGAGCATTCCTTCGAGGTTCCAACCTACGCTGAGACCCTAGACGAAGCTCTGGAGTTAGCCGAATGGCAATACGTTCCGGCTGGCTTTGAGGTTACTCGTGTGCGTCCTTGTGTAGCACCGAAGTAATACGACTCACTATTAGGGAAGACTCCCTCTGAGAAACCAAACGAAACCTAAAGGAGATTAACATTATGGCTAAGAAGATTTTCACCTCTGCGCTGGGTACCGCTGAACCTTACGCTTACATCGCCAAGCCGGACTACGGCAACG   >  1:25542/1‑301`  
                                                                                                                                                                                                                                                                                                                  `TAAGAAGTTTTGGGCTACCGTAGAGTCCTCGGAGCATTCCCTCGAGGTTCCAATCTACGCTGAGACCCTAGACGAAGCTCTGGAGTTAGCCGAATGGCAATACGTTCCGGCTGGCTTTGAGGTTACTCGTGTGCGTCCTTGTGTAGCACCGAAGCAATACGACTCACTATTAGGGAAGACTCCCTCTGAGAAACCAAACGAAACCTAAAGGAGATTAACATTATGGCTAAGAAGATTTTCACCTCTGCGCTGGGTACCGCTGAACCTTACGCTTACATCGCCTAGCCGGACTCCGGCAACG   >  1:558011/1‑301`  
                                                                                                                                                                                                                                                                                                                   `AAGAAGTTTTGGTCTACCGTAGAGTCCTCGGAGCATTCCTTCGAGGTTCCAATCTACGCTGAGACCCTAGACGAAGCTCTGGAGTTAGCCGAATGGCAATACGTTCCGGATGGCTTTGAGGTTACTCGTGTGCGTCCTTGTGTAGCACCGAAGTAATACGACTCACTATTAGGGAAGACTCCCTCTGAGAAACCAAACGAAACCTAAAGGAGATTAACATTATGGCTAAGAAGATTTTCACCTCTGCGCTGTGTACCGCTGAACCTTACGCTTACATCGCCAAGCCGGACTACGGCAACGA  <  1:145514/301‑1`  
                                                                                                                                                                                                                                                                                                                   `AAGAAGTTTTGGGCTACCGTAGAGTCCTCGGAGCATTCCTTCGAGGTTCCAATCTACGCTGAGACCCTAGACGAAGCTCTGGAGTTAGCCGAATGGCAATACGTTCCGGCTGGCTTTGAGGTTACTCGTGTGCGTCCTTGTGTAGCACCGAAGTAATACGACTCACTATTAGGGAAGACTCCCTCTGAGAAACCAAACGAAACCTAAAGGAGATTAACATTATGGCTAAGAAGATTTTCACCTCTGCGCTGGGTACCGCTGAACCTTACGCTTACATCGCCAAGCCGGACTACGGCAACGA  <  1:608479/301‑1`  

                                                                                                                                                                                                                                                                                                                                                                                                                                                                                                                                                                                                                                  
`GGCACCGAGCGTCCACATCATTATCACCCGTGGTGACTTTGAGAAAGCAATAGACAAGAAACGTCCGGTTCTTAGTGTGGCAGTGACCCGCTTCCCGTTCGTCCGTCTGTTACTCAAACGAATCAAGGAGGTGTTCTGATGGGACTGTTAGATGGTGAAGCCTGGGAAAAAGAAAACCCGCCAGTACAAGCAACTGGGTGTATAGCTTGCTTAGAGAAAGATGACCGTTATCCACACACCTGTAACAAAGGAGCTAACGATATGACCGAACGTG‑AACAAGAGATGATCATTAAGTTGATAGACAATAA‑‑‑‑‑‑‑‑‑‑‑‑‑‑‑‑‑‑‑‑‑‑‑‑‑‑‑‑‑‑‑‑‑‑‑‑‑‑‑‑‑‑‑‑‑‑‑‑‑‑‑‑‑‑‑‑‑‑‑‑‑‑‑‑‑‑‑‑‑‑‑‑‑‑‑‑‑‑‑‑‑‑‑‑‑‑‑‑‑‑‑‑‑‑‑‑‑‑‑‑‑‑‑‑‑‑‑‑‑‑‑‑‑‑‑‑‑‑‑‑‑‑‑‑‑‑‑‑‑‑‑‑‑‑‑‑‑‑‑‑‑‑‑‑‑‑‑‑‑‑‑‑‑‑‑‑‑‑‑‑‑‑‑‑‑‑‑‑‑‑‑‑‑‑‑‑‑‑‑‑‑‑‑‑‑‑‑‑‑‑‑‑‑‑‑‑‑‑‑‑‑‑‑‑‑‑‑‑‑‑‑‑‑‑‑‑‑‑‑‑‑‑‑‑‑‑‑‑‑‑‑‑‑‑‑‑‑‑‑‑‑‑‑‑‑‑‑‑‑‑‑‑‑‑‑‑‑‑‑‑‑‑‑‑‑‑‑‑‑‑‑‑‑‑‑‑‑‑‑‑‑‑‑‑‑‑‑‑‑‑‑‑‑‑‑‑‑‑‑  >  NC_001604.1/8028‑8335`  
`‑‑‑‑‑‑‑‑‑‑‑‑‑‑‑‑‑‑‑‑‑‑‑‑‑‑‑‑‑‑‑‑‑‑‑‑‑‑‑‑‑‑‑‑‑‑‑‑‑‑‑‑‑‑‑‑‑‑‑‑‑‑‑‑‑‑‑‑‑‑‑‑‑‑‑‑‑‑‑‑‑‑‑‑‑‑‑‑‑‑‑‑‑‑‑‑‑‑‑‑‑‑‑‑‑‑‑‑‑‑‑‑‑‑‑‑‑‑‑‑‑‑‑‑‑‑‑‑‑‑‑‑‑‑‑‑‑‑‑‑‑‑‑‑‑‑‑‑‑‑‑‑‑‑‑‑‑‑‑‑‑‑‑‑‑‑‑‑‑‑‑‑‑‑‑‑‑‑‑‑‑‑‑‑‑‑‑‑‑‑‑‑‑‑‑‑‑‑‑‑‑‑‑‑‑‑‑‑‑‑‑‑‑‑‑‑‑‑‑‑‑‑‑‑‑‑‑‑‑‑‑‑‑‑‑‑‑‑‑‑‑‑‑‑‑‑‑‑‑‑‑‑‑‑‑‑‑‑‑‑‑‑‑‑‑‑‑‑‑‑‑‑‑‑‑‑‑‑‑‑‑‑‑‑‑‑‑‑‑‑‑‑‑‑‑‑‑‑‑‑‑gacaataaGAAGTTTTGGGCTACCGTAGAGTCCTCGGAGCATTCCTTCGAGGTTCCAATCTACGCTGAGACCCTAGACGAAGCTCTGGAGTTAGCCGAATGGCAATACGTTCCGGCTGGCTTTGAGGTTACTCGTGTGCGTCCTTGTGTAGCACCGAAGTAATACGACTCACTATTAGGGAAGACTCCCTCTGAGAAACCAAACGAAACCTAAAGGAGATTAACATTATGGCTAAGAAGATTTTCACCTCTGCGCTGGGTACCGCTGAACCTTACGCTTACATCGCCAAGCCGGACTACGGCAACGA  >  NC_001604.1/8939‑9237`

  

Alignment Legend

Aligned base mismatch/match (shaded by quality score): `ATCG/ATCG` `< 3 ≤` `ATCG/ATCG` `< 10 ≤` `ATCG/ATCG` `< 23 ≤` `ATCG/ATCG` `< 35 ≤` `ATCG/ATCG` `< 38 ≤` `ATCG/ATCG`

Unaligned base: `atcg`    Masked matching base: `atcg`    Alignment gap: `‑`    Deleted base: `‑`

Reads not counted as support for junction

read\_name Not counted due to insufficient overlap past the breakpoint.

read\_name Not counted due to not crossing MOB target site duplication.
